# Development and cross-validation of LMS-based normative reference standards and health benefits zones for muscular strength among adolescents by age and sex

**DOI:** 10.3389/fpubh.2025.1616298

**Published:** 2025-08-26

**Authors:** Yang Yang, Syed Ghufran Hadier, Liu Long, Syed Muhammad Zeeshan Haider Hamdani, Syed Danish Hamdani

**Affiliations:** ^1^School of Physical Education, Suzhou University, Suzhou, Anhui, China; ^2^Department of Sports Sciences, Bahauddin Zakariya University, Multan, Pakistan; ^3^School of Physical Education, Shanxi University, Taiyuan, China; ^4^School of Exercise and Health, Shanghai University of Sport, Shanghai, China; ^5^Division of Olympic Sports, China Swimming College, Beijing Sport University, Beijing, China

**Keywords:** muscular strength, handgrip strength, normative reference standards, health benefit zones, LMS method, back-generation method, adolescent fitness, cross-validation

## Abstract

**Objective:**

This study aims to develop and validate age- and sex-specific normative reference standards for muscular strength (MS) using the LMS (Lambda–Mu–Sigma) method and to establish Health Benefit Zones (HBZs) for Pakistani adolescents aged 12–16 years.

**Methods:**

A cross-sectional study was conducted with 2,970 adolescents (49.7% boys, 50.3% girls) selected through stratified random sampling from 60 public high schools across three divisions of South Punjab. Anthropometric indicators and muscular strength were measured following standardized protocols. Using the LMS, age- and sex-specific normative reference values and smoothed percentile curves (3rd, 10th, 35th, 50th, 65th, and 90th) were developed. Five Health Benefit Zones (Very Poor, Poor, Medium, Good, and Excellent) were derived from these percentile ranges to classify strength levels. The robustness of the generated standards was examined through internal cross-validation using a back-generation procedure to confirm high predictive accuracy.

**Results:**

Boys demonstrated significantly higher muscular strength than girls across all ages (*p* < 0.001), with strength increasing progressively with age in both sexes. At age 16, median MS reached 35.47 kg for boys and 20.18 kg for girls. LMS-derived percentile reference values and percentile curves illustrated consistent age- and sex-related growth trends. Approximately 40% of participants fell within the “poor” or “very poor” HBZs. MAPE values remained below ±0.05, indicating excellent model fit. Compared to international benchmarks, adolescents from South Punjab exhibited lower MS values across corresponding age groups.

**Conclusion:**

This study provides the first LMS-based, age- and sex-specific normative reference standards and HBZs for muscular strength among Pakistani adolescents. These standards offer a population-relevant tool for fitness assessment, enable early identification of youth at risk of low muscular strength, and support targeted interventions to enhance strength development and overall physical health in school-aged populations.

## 1 Introduction

Muscular strength is a key component of health-related fitness and a critical marker of growth, functional ability, and long-term health in youth (1). Among various strength indicators, handgrip strength (HGS) is widely recognized for its practicality, reliability, and relevance in field-based assessments (2, 3). As a simple, non-invasive measure, HGS offers valuable insights into overall muscular fitness, nutritional status, and physical development in children and adolescents (4).

There is growing evidence that muscular strength during adolescence is associated with a wide range of physical and metabolic health outcomes, including bone health, cardiovascular function, and psychosocial well-being (4, 5). Conversely, low muscular fitness has been linked to poor metabolic profiles, increased adiposity, reduced functional capacity, and heightened risk of chronic disease in adulthood (5, 6). These associations underscore the importance of incorporating muscular fitness assessment into school-based health surveillance and national fitness monitoring systems (7).

In both clinical and public health contexts, HGS serves as a practical screening tool for detecting early developmental delays and suboptimal physical growth (8). Early identification of low muscular strength can help guide preventive strategies and improve long-term health trajectories (9, 10). Consequently, accurate, population-specific normative reference values are essential for identifying deviations from typical development and informing early interventions (11, 12).

Over the past two decades, several countries have established age- and sex-specific normative values for handgrip strength in youth populations, primarily in high-income regions such as the United States, Europe, Australia, and East Asia (China and South Korea) (11, 13–17). These studies consistently show that HGS increases with age and displays marked sex differences during and after puberty (2). However, there is considerable inter-population variability influenced by ethnicity, nutritional status, lifestyle behaviors, and socio-economic conditions (18, 19). Consequently, applying reference values from other populations may lead to misclassification and inaccurate health risk assessments, particularly in low- and middle-income countries (LMICs) undergoing nutritional transitions.

Comparative research has shown that adolescents from developing regions often present with lower HGS than their counterparts in more affluent settings. For example, Rostamzadeh et al. (2) found that Iranian adolescents had significantly lower grip strength values than those from Europe and North America, especially after age 12. These findings emphasize the need for population-specific reference standards to avoid misclassification and ensure culturally and biologically relevant health assessments.

Despite the growing body of global data, there remains a paucity of HGS reference standards for youth in South Asia and other developing regions. Pakistan, in particular, has lacked comprehensive normative values for muscular strength in its adolescent population (20). South Punjab, a region characterized by economic disadvantages and limited access to youth fitness programs has no published reference standards. This absence is problematic because applying reference norms from other countries may misclassify the strength status of Pakistani children. Establishing localized norms is therefore crucial for effective health monitoring, clinical diagnosis, and policy planning (14, 21). From a public health perspective, having baseline strength distributions for Pakistani adolescents allows authorities to identify subgroups with low muscular fitness and track improvements in response to nutrition or physical activity interventions (22). Generating indigenous reference standards is thus a high priority to inform youth fitness assessment and intervention programs in regions like South Punjab.

The present study addresses this critical gap by establishing and internally validating age- and sex-specific normative reference standards for absolute handgrip strength among adolescents aged 12–16 years in South Punjab, Pakistan. Using the Lambda–Mu–Sigma (LMS) method, smoothed percentile curves were generated, and the back-substitution method was employed for internal cross-validation. In addition to producing normative values, this study also aimed to develop Health Benefit Zones (HBZs) for muscular strength, providing a practical framework for classifying strength levels and identifying individuals at increased health risk. Together, these standards offer an evidence-based tool for educators, clinicians, and policymakers to assess muscular fitness, monitor trends, and implement targeted health and fitness interventions tailored to the needs of this specific population.

## 2 Methods

This cross-sectional study was conducted during the 2019 academic year, to evaluate absolute muscular strength among adolescents in South Punjab, Pakistan using a stratified random sampling technique. As part of the Young Teen’s Assessment Active Lifestyle Involvement—PAKistan Study (YAALI-PAK) (23). South Punjab served as the primary stratum and was further divided into three sub-strata: Multan, Bahawalpur, and Dera Ghazi Khan divisions. These regions provide a representative sample of the South Punjab adolescent population (24, 25). South Punjab province comprises 360 higher secondary schools (20). Using an equal allocation method, 20 schools were randomly selected from each sub-stratum, totaling 60 schools (16.67% of the total).

### 2.1 Sample size

The sample size was estimated using the standard formula: $n=\frac{{\left(Z\right)}^{2}\mathrm{PQ}}{e^{2}}\times D$ (26), where *P* denotes the estimated population proportion (0.40), *Q* its complement (0.60), *Z* the z-score for a 95% confidence level (1.96), *e* the margin of error (0.05), and *D* the design effect (1.0). This calculation yielded a minimum required sample size of 369 participants. To enhance generalizability and support the development of robust normative reference standards, a larger sample of 3,000 adolescents aged 12–16 years was targeted. This sample size ensured over 80% statistical power at a 5% significance level and was validated using standard sample size calculation tools (27). More detailed information regarding the sampling approach is available in a previously published study (23).

Utilizing a stratified random sampling strategy with an equal allocation method, participants were recruited from 60 schools, with 50 students selected per school, with 10 students per age group (12 to 16 years). To ensure gender balance, each age group included five boys and five girls, resulting in 25 boys and 25 girls per school. After excluding approximately 1% of cases due to incomplete data, the final analytic sample comprised 2,970 participants (49.73% boys and 50.26% girls). This design resulted in approximately 595 participants in each age group, with nearly equal about 20% representation of boys and girls (approximately 295 in each group), ensuring proportional and unbiased distribution across all subgroups.

### 2.2 Inclusion and exclusion criteria

Participants were healthy adolescents with no history of physical disabilities, cognitive impairments, clinical conditions, surgeries, or ongoing medication. Eligibility was confirmed through school medical records and physical examinations. Individuals with any musculoskeletal, medical, or cognitive conditions affecting assessments were excluded. Participation was voluntary, with the right to withdraw at any time. These criteria ensured a valid, reliable sample for generalizable findings.

### 2.3 Ethical approval

This study received ethical approval from two institutions: the School of Exercise and Health at Shanghai University of Sport in September 2018 (Approval No. 1716516032) and the University Research Ethics Committee of Bahauddin Zakariya University, Multan (Approval No. 374/UREC). Prior to data collection, written and verbal consent was obtained from educational authorities, school administrators, and parents to ensure transparency and voluntary participation. The research strictly followed institutional and international ethical standards to safeguard participant rights, dignity, and confidentiality. All procedures were conducted with full approval and oversight, ensuring that the study met the highest standards of ethical conduct in both countries.

### 2.4 Data collection procedures

Before initiating data collection, we obtained formal approvals from the Education Department and school administrations, along with verbal parental consent and informed consent from school principals, in accordance with ethical standards for research involving minors. A team of 12 research assistants from the Department of Sports Sciences at Bahauddin Zakariya University were recruited and trained through a structured workshop. The training emphasized adherence to standardized testing protocols, proper use of equipment, and accurate data collection procedures.

Data collection was conducted during the 2019 academic year across schools in South Punjab, Pakistan. The testing schedule was coordinated with each school’s timetable. The test team contacted the focal personnel at the sampled schools to confirm class availability, communicated the test requirements 2 days in advance, and visited the school on the designated day. On the day of testing, a random selection of students aged 12–16 were drawn from the official school list, and these students were included in the final sample. Class teachers ensured their attendance for subsequent visits. On the first day, anthropometric assessments were conducted, including measurements of height, weight and BMI calculation. On the second day, muscular strength was evaluated using the handgrip strength test. All measurements were completed during regular school hours across two consecutive visits per school.

### 2.5 Measures

Basic demographic information, including participants’ age and sex, was obtained either through self-report or extracted from official school records.

#### 2.5.1 Body measurement

Body measurement were conducted with participants standing barefoot, ensuring the head and shoulders were positioned at a right angle to the measuring scale by following international protocols of the Center for Disease Control, CDC, USA, 2012 (28).

Height was assessed in centimeters (cm) as a key indicator of physical growth and development during adolescence. Measurements were obtained using a height–weight scale (DT-150 Height and Weight Scales, Shanghai, China), with the integrated metric rod used to determine stature. Participants stood upright on a level surface with their backs against a vertical plane, heels together, toes pointing outward at approximately a 60-degree angle, and arms resting naturally at their sides. Height values were recorded to the nearest 0.1 cm to ensure measurement precision.

Body weight was recorded in kilograms (kg), serving alongside height to determine BMI status. The same height–weight scale (DT-150 Height and Weight Scales, Shanghai, China) was used for this purpose. Participants stood barefoot and balanced on the scale during the measurement. Weight values were recorded to one decimal point in kilograms.

Body Mass Index (BMI) was computed by dividing body weight (in kilograms) by the square of height (in meters) as following: BMI=Weight (kg)/Height (m)^2^. This index is widely recognized as a standardized method for assessing weight-related health risks and categorizing weight status across populations. It enables neutral and consistent comparisons by accounting for height variations among individuals.

### 2.6 Muscular strength

Muscular strength was evaluated using the handgrip strength test, a validated and widely accepted measure of overall muscular strength in adolescents (29). An electronic dynamometer (GRIPX Digital Hand Dynamometer) was used for measurement. Before testing, participants engaged in light stretching, as prior stretching has been shown to mitigate reductions in peak force (9). A standardized familiarization trial (5–10 s) was conducted with clear verbal instructions to ensure procedural understanding. The grip span of the dynamometer was adjusted for each participant so that the second metacarpophalangeal joint aligned optimally with the handle, facilitating maximal force application. The device was reset to “0.00” prior to each trial to ensure accurate recordings.

Participants stood in an upright posture, feet shoulder-width apart, with the test arm extended downward at an angle of approximately 30 ° from the torso. They were instructed to squeeze the dynamometer with maximal effort for at least 3 s. Standardized verbal encouragement was provided during each trial to motivate peak performance. The device automatically recorded the highest force generated during the isometric contraction. Each hand was tested three times in an alternating sequence, with 30-s rest intervals between trials to minimize fatigue. The highest force output from each hand (in kilograms, to the nearest 0.1 kg) was recorded.

Absolute handgrip strength was calculated as the average of the maximal values from the right and left hands (30, 31), consistent with established adolescent muscular strength assessment protocols (3, 32, 33). A higher score reflected greater overall muscular performance.

### 2.7 Healthy benefit zones

Health Benefit Zones (HBZs), comprising five categories Very Poor, Poor, Medium, Good, and Excellent were established based on absolute HGS percentiles for boys and girls aged 12–16 years. These zones provide an evidence-based classification system for evaluating muscular strength in adolescents, aligning with age- and sex-specific percentile thresholds.

The categorization methodology is informed by previous normative frameworks used in Asian and European populations, employing a five-tier percentile-based scoring model (34–36). Specifically, the thresholds were defined as follows: Very Poor (<P3), Poor (P3–P10), Medium (P10–P50), Good (P50–P90), and Excellent (≥P90). These cut-offs align with established reference systems, including the China National Fitness Standards (2014), which intentionally employ unequal percentile bands to better capture developmental variability, enhance diagnostic sensitivity, and improve their utility for performance evaluation and health risk identification (35, 37).

### 2.8 Statistical analysis

A quantitative research design was employed, incorporating both descriptive and inferential statistical procedures. Descriptive statistics including frequencies, percentages, means, and measures of dispersion were calculated. The results are presented as medians and interquartile ranges (IQR) were reported. Differences in HGS across sex and age were further assessed using a two-way analysis of variance (ANOVA) to examine main effects and interaction effects (sex × age). The two-way ANOVA was applied due to its robustness in large samples (*n* > 1,000), as supported by statistical literature (38). In addition, scatterplots with age-specific regression trendlines were generated separately for boys and girls to illustrate the progression of HGS in relation to body mass, height, and BMI across the different age groups.

To construct smoothed, age- and sex-specific reference percentiles for handgrip strength, the LMS (Lambda–Mu–Sigma) method was applied using the *gamlss* package in R. We employ Box–Cox transformations, median smoothing, and coefficient of variation adjustments within the LMS method to construct smoothed, age- and sex-specific normative reference curves. This technique estimates three parameters: Lambda (*λ*, skewness), Mu (*μ*, median), and Sigma (*σ*, coefficient of variation), enabling the generation of age-adjusted percentile curves (P3, P10, P50, P90, P97) that account for non-normality and developmental changes across age groups (39).

### 2.9 Back-generation testing for validation of normative reference standards

To validate the developed reference standards, an internal cross-validation was conducted using a back-generation test (20). This involved generating 50th percentile (P50) values from randomly selected holdout datasets stratified by age and sex (20, 23). Predictive accuracy was evaluated using the Mean Absolute Percentage Error (MAPE), which quantifies the average deviation between observed and predicted values. Based on Lewis’s classification (Table 1), MAPE values below 10% denote “high accuracy,” 10–20% “good,” 20–50% “reasonable,” and above 50% “inaccurate” (40). The consistently low MAPE values observed across subgroups demonstrated high predictive validity and reliability of the established normative reference standards, confirming the methodological rigor of the evaluation system.

**Table 1:** Interpretation criteria of mean absolute percentage error (MAPE).

MAPE categories	Interpretation/forecasting power
<10	Highly accurate forecasting
10–20	Good forecasting
20–50	Reasonable forecasting
>50	Inaccurate forecasting

## 3 Results

In the present study, 2,970 adolescents aged 12–16 years were included, comprising 1,477 boys (49.7%) and 1,493 girls (50.3%). As shown in Table 2, each age group contained approximately 595 participants with near-equal representation of boys and girls (e.g., age 12: 291 boys, 299 girls; age 16: 295 boys, 300 girls), demonstrating a balanced distribution across both age and gender strata. Table 3 presents the gender-specific medians and interquartile ranges (IQRs) for anthropometric and absolute muscular strength variables. The median age of the participants was 14 years (IQR = 2), with no statistically significant difference between boys and girls (*p* = 0.927). The median height of the total sample was 159.00 cm (IQR = 11.00), with boys being significantly taller than girls (160.00 cm [IQR = 13.00] vs. 158.00 cm [IQR = 10.00], *p* < 0.001). Similarly, boys had a significantly higher median body weight compared to girls (45.02 kg [IQR = 11.00] vs. 41.00 kg [IQR = 10.00], *p* < 0.001). The overall median BMI was 16.80 kg/m^2^ (IQR = 2.67), with boys showing a significantly higher median BMI than girls (17.29 kg/m^2^ [IQR = 2.82] vs. 16.02 kg/m^2^ [IQR = 3.44], *p* < 0.001). Regarding muscular strength, the median absolute handgrip strength for the total sample was 21.30 kg (IQR = 14.00). Boys demonstrated significantly higher median grip strength (31.88 kg [IQR = 15.00]) compared to girls (17.06 kg [IQR = 5.39]), nearly double on average (*p* < 0.001).

**Table 2:** Age- and gender-specific differences in height (cm), weight (kg), BMI, or handgrip strength (kg) based on ANOVA.

Age	Gender	Height (cm)		Weight (kg)		BMI		HGS (kg)	
		Median (IQR)	ANOVA (Gender)	Median (IQR)	ANOVA (Gender)	Median (IQR)	ANOVA (Gender)	Median (IQR)	ANOVA (Gender)
12	Boys (*n* = 291)	149.00 (14.00)	*F* = 5.410 *p* = <0.05 *η*^2^ = 0.009	35.55 (13.00)	*F* = 2.413 *p* = 0.121 *η*^2^ = 0.004	16.44 (2.70)	*F* = 8.477 *p* = <0.001 *η*^2^ = 0.014	21.80 (17.80)	*F* = 151.396 *p* = <0.001 *η*^2^ = 0.205
	Girls (*n* = 299)	153.00 (14.00)		35.50 (11.00)		15.62 (4.20)		13.60 (6.10)	
13	Boys (*n* = 295)	159.00 (15.00)	*F* = 0.086 *p* = 0.769 *η*^2^ = 0.000	43.00 (12.80)	*F* = 48.359 *p* = <0.001 *η*^2^ = 0.076	16.75 (2.50)	*F* = 63.505 *p* = <0.001. *η*^2^ = 0.097	27.60 (11.90)	*F* = 332.480 *p* = <0.001 *η*^2^ = 0.360
	Girls (*n* = 298)	158.00 (14.00)		38.00 (9.00)		15.23 (3.56)		15.10 (6.20)	
14	Boys (*n* = 298)	165.00 (11.00)	*F* = 12.965 *p* = <0.001 *η*^2^ = 0.021	48.00 (9.63)	*F* = 69.462 *p* = <0.001 *η*^2^ = 0.105	17.63 (2.48)	*F* = 43.291 *p* = <0.001 *η*^2^ = 0.068	30.80 (15.20)	*F* = 347.175 *p* = <0.001 *η*^2^ = 0.370
	Girls (*n* = 295)	162.00 (8.00)		40.00 (8.75)		15.62 (2.83)		16.80 (7.70)	
15	Boys (*n* = 298)	165.00 (9.00)	*F* = 31.106 *p* = <0.001 *η*^2^ = 0.021	44.83 (7.25)	*F* = 13.101 *p* = <0.001 *η*^2^ = 0.022	16.22 (2.13)	*F* = 0.097 *p* = 0.755 *η*^2^ = 0.000	30.85 (9.10)	*F* = 315.635 *p* = <0.001 *η*^2^ = 0.346
	Girls (*n* = 300)	162.00 (10.00)		43.50 (9.00)		16.36 (3.43)		19.10 (8.10)	
16	Boys (*n* = 295)	167.00 (12.00)	*F* = 65.136 *p* = <0.001 *η*^2^ = 0.099	49.00 (10.00)	*F* = 118.825 *p* = <0.001 *η*^2^ = 0.167	17.60 (3.75)	*F* = 46.905 *p* = <0.001 *η*^2^ = 0.073	36.10 (13.60)	*F* = 373.587 *p* = <0.001 *η*^2^ = 0.387
	Girls (*n* = 300)	162.00 (8.00)		44.00 (8.00)		16.53 (3.02)		20.60 (8.50)	
Total	Age × Gender	164.61 ± 7.96	*F* = 14.14 *p* = <0.001 *η*^2^ = 0.019	47.36 ± 7.99	*F* = 11.63 *p* = <0.001 *η*^2^ = 0.015	17.44 ± 2.34	*F* = 8.85 *p* = <0.001 *η*^2^ = 0.012	29.99 ± 15.64	F = 21.55 *p* = <0.001 *η*^2^ = 0.028

The data was presented as median ± Interquartile Range. *N*: numbers. HGS (kg): Handgrip Strength (kilograms). F and *p* values reflect the results of one-way ANOVA comparing boys and girls within each age group. Partial eta squared (*η*^2^) represents the effect size for gender.

**Table 3:** Gender specific anthropometric and muscular strength of participants.

Component	Total (2970) Median (IQR)	Boys (1477) Median (IQR)	Girls (1493) Median (IQR)	*p*-value
Age (years)	14.00 (2.00)	14.00 (2.00)	14.00 (2.00)	0.927
Height (cm)	161.00 (13.00)	163.00 (13.00)	160.00 (12.00)	<0.001
Weight (kg)	43.00 (11.00)	45.00 (11.85)	41.00 (10.00)	<0.001
BMI (kg/m2)	16.45 (3.07)	16.81 (2.67)	16.02 (3.34)	<0.001
HGS (kg)	21.30 (14.00)	29.60 (14.20)	16.80 (8.20)	<0.001

The data was presented as median ± Interquartile Range. HGS (kg): Handgrip Strength (kilograms).

Table 2 presents age- and gender-specific descriptive statistics for handgrip strength among adolescents aged 12–16 years. A consistent age-related increase in HGS was observed in both boys and girls, with statistically significant differences between sexes across all age groups (*p* < 0.001). For boys, median HGS rose from 21.80 kg (IQR = 17.80) at age 12 to 36.10 kg (IQR = 13.60) at age 16. An increase was noted between ages 13 and 14, with median values of 27.60 kg (IQR = 11.90) and 30.80 kg (IQR = 15.20), respectively, followed by continued increases through ages 15 and 16. For girls, HGS increased steadily from 13.60 kg (IQR = 6.10) at age 12 to 20.60 kg (IQR = 8.50) at age 16, without any notable declines. At every age, boys consistently outperformed girls, and all sex-based differences were significant (*p* < 0.001). A two-way ANOVA showed significant main effects of gender (*F* = 1489.35, *η*^2^ = 0.335, *p* < 0.001) and age (*F* = 92.23, *η*^2^ = 0.111, *p* < 0.001), along with a significant gender × age interaction (*F* = 21.55, *η*^2^ = 0.028, *p* < 0.001). These findings confirm that both age and sex meaningfully influence handgrip strength during adolescence, with boys demonstrating higher absolute strength and greater gains across the studied age range.

**Figure 1:**
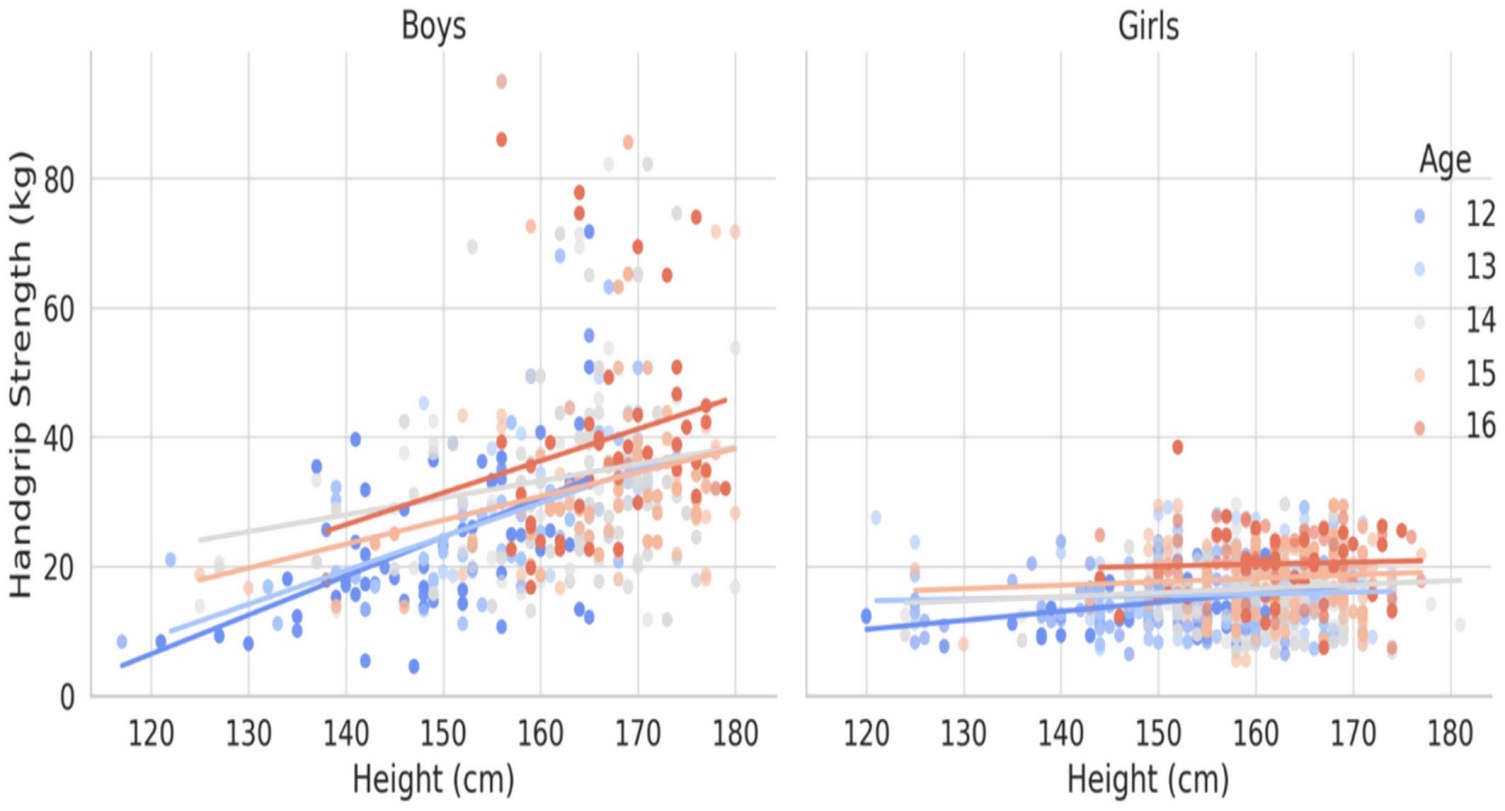
HGS (kg) percentile curve for the South Punjab.

**Figure 2:**
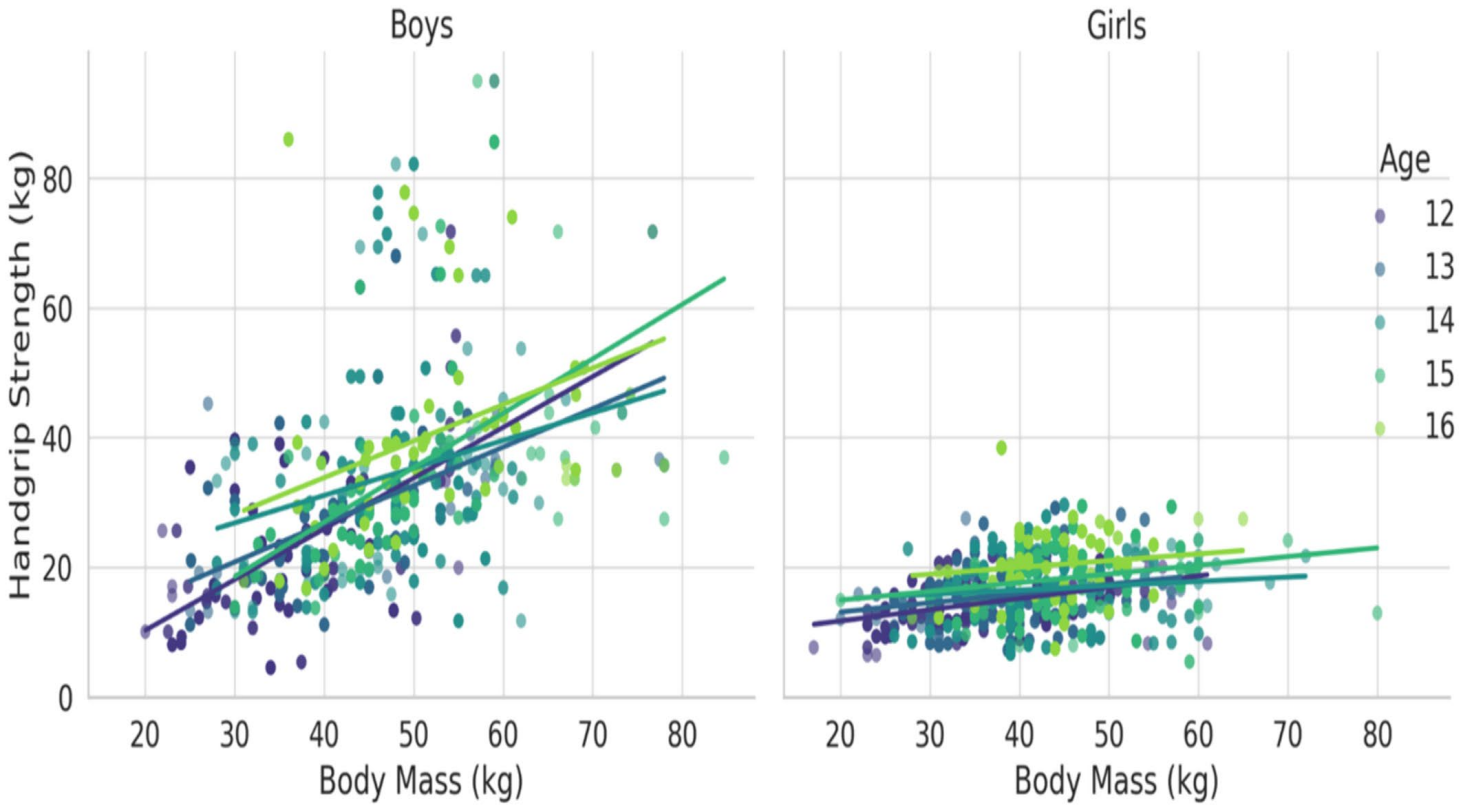
HGS (kg) percentile curve for the South Punjab.

**Figure 3:**
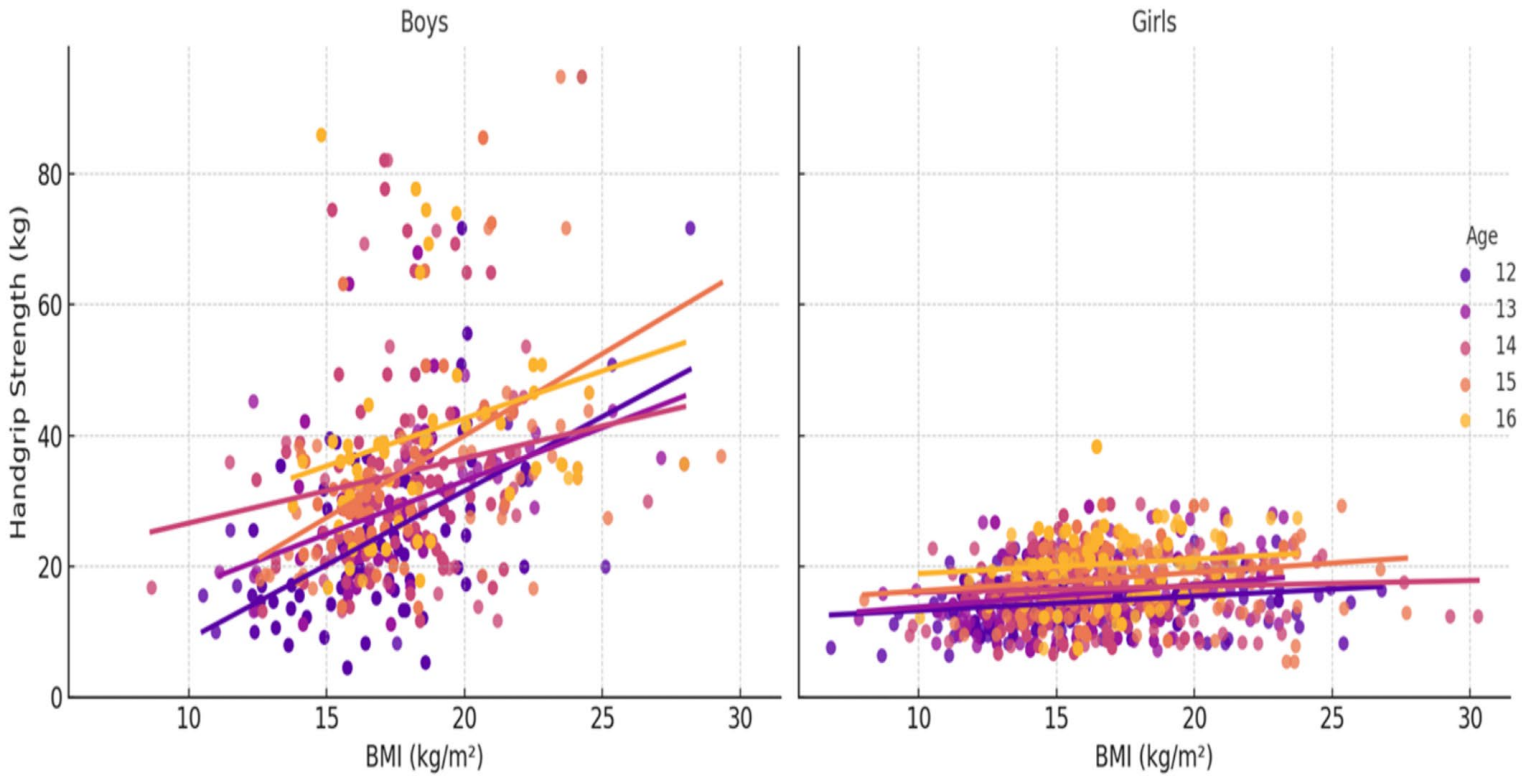
HGS (kg) percentile curve for the South Punjab.

Figures 1–3 illustrate the age- and gender-specific progression of absolute handgrip strength (HGS) in relation to height, body mass, and BMI, respectively, for adolescents from South Punjab. As shown in Figure 1, HGS increased progressively with height in both boys and girls. Boys demonstrated consistently higher HGS values than girls across all age groups. The slopes of the regression lines indicate that older age groups (15–16 years) exhibited steeper gains in HGS per unit increase in height compared with younger groups, particularly in boys.

In Figure 2, a similar trend was observed with body mass. HGS increased markedly with increasing body mass across all age groups, with boys again exhibiting higher absolute HGS than girls. The separation of age-specific trendlines shows that older adolescents, especially boys aged 15–16 years, demonstrated substantially greater strength for a given body mass compared with their younger counterparts.

Figure 3 depicts HGS in relation to BMI. HGS generally increased with BMI, but the association was less pronounced than with body mass or height. Nevertheless, the interaction between age and gender remained evident: older boys displayed the highest HGS values across the BMI, whereas girls showed comparatively smaller increments in HGS with increasing BMI.

### 3.1 Back- generation test hand grip (kg)

In our study, we used the back generation test to evaluate the cross-validation of normative standard for HGS. The current study involved randomly extracting a small data set to compare P 50th values of the large (actual value) and small (fitted values), using the LMS method to establish the normative standard. By assessing the degree of coincidence between data sets generated through random sampling, we ensured the credibility and applicability of normative standard. Table 4 displays the actual and fitted median values of HGS (in kilograms) by age and gender, along with the mean absolute percentage error (MAPE) for each group. Among boys, MAPE values ranged from −0.03 to 0.06, while for girls, MAPE values varied between −0.05 and 0.10. On average, the MAPE was −0.007 for males and 0.012 for females, indicating a high level of accuracy in the normative model. These results confirm that the differences between actual and predicted values are within acceptable margins of error, thereby supporting the reliability and robustness of the back-generated normative standards. The low MAPE values across both sexes and all age groups underscore the precision of the LMS-derived norms.

**Table 4:** Age and gender-specific back generation testing of HGS (kg).

Age	Male			Female		
	Actual value	Fitted value	MAPE	Actual value	Fitted value	MAPE
12	22.53	23.20	−0.03	14.12	13.95	0.01
13	25.77	25.00	0.03	15.01	14.50	0.04
14	29.00	29.55	−0.02	16.48	14.95	0.10
15	32.24	31.10	0.04	18.16	19.10	−0.05
16	35.47	37.50	−0.05	20.18	21.00	−0.04
Average			−0.007			0.012

MAPE: mean absolute percentage error.

### 3.2 Reference values and centile curves

Table 5 presents the smoothed LMS percentile values (3rd, 10th, 35th, 50th, 65th, and 90th) for handgrip strength (HGS) among adolescents aged 12 to 16 years in South Punjab. These values, derived using the LMS method, provide age- and gender-specific normative reference standards based on the distribution’s skewness (L), median (M), and variability (S). The 50th percentile (median) values demonstrate a clear upward trend in handgrip strength with age in both boys and girls. Among boys, the median HGS increased from 22.53 kg at age 12 to 35.47 kg at age 16, representing an overall increase of 12.94 kg over 4 years, or an average annual increase of approximately 3.24 kg/year. For girls, the median increased from 14.12 kg at age 12 to 20.23 kg at age 16, reflecting a total gain of 6.11 kg and an average annual increase of approximately 1.53 kg/year.

**Table 5:** HGS (kg) percentile by age and gender in adolescents aged 12–16 from South Punjab.

Percentile	L	S	3	10	35	M 50	65	90
Boys								
12	0.227	0.483	8.14	11.56	18.63	22.53	27.03	40.21
13	−0.004	0.432	11.45	14.82	21.82	25.77	30.43	44.86
14	−0.198	0.393	14.55	17.94	24.98	29.00	33.82	49.31
15	−0.339	0.364	17.42	20.90	28.11	32.24	37.22	53.58
16	−0.413	0.345	19.96	23.63	31.17	35.47	40.66	57.81
Girls								
12	0.268	0.279	8.02	9.59	12.66	14.12	15.70	19.87
13	0.442	0.317	7.51	9.69	13.24	15.01	16.90	21.81
14	0.615	0.331	7.52	10.08	14.43	16.48	18.63	24.03
15	0.789	0.293	8.81	11.63	16.14	18.16	20.23	25.23
16	0.963	0.286	9.46	12.84	17.96	20.18	22.41	27.62

*N*, Number of participants; M, median.

These results indicate that boys consistently outperform girls in grip strength across all percentiles and ages, with a notably steeper rate of increase. The largest gains for both sexes were observed between ages 14 and 15. Additionally, higher percentiles (90th) showed even greater differences, emphasizing variability in strength development, especially among boys. Overall, the LMS-derived percentiles provide strong evidence of age- and sex-specific progression in muscular strength, with boys exhibiting both higher levels and steeper improvements in handgrip strength during early to mid-adolescence.

**Figure 4:**
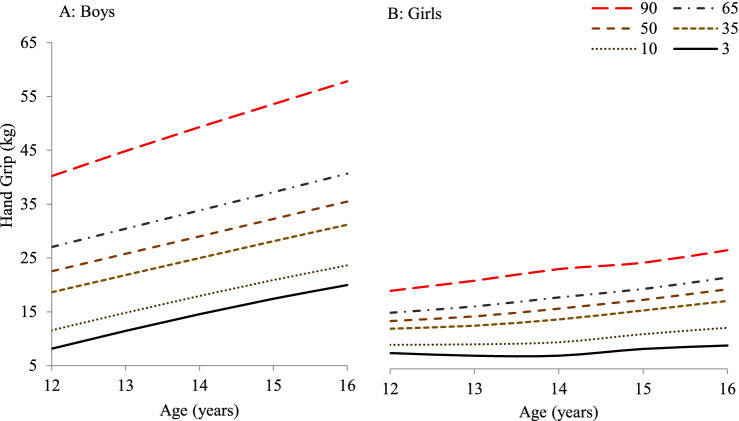
HGS (kg) percentiles (3rd to 90th) for adolescent boys **(A)** and girls **(B)** aged 12–16 years from the present study.

Figure 4 illustrates the smoothed percentile curves for HGS among boys and girls aged 12–16 years in South Punjab, corresponding to the numerical values presented in Table 5. The percentiles plotted include the 3rd, 10th, 35th, 50th (median), 65th, and 90th percentiles, offering a comprehensive view of strength distribution and growth trends across adolescence. In Panel A (Boys), the curves show a consistent and pronounced increase in HGS with age across all percentiles. The 50th percentile (dashed brown line) progresses from approximately 22.53 kg at age 12 to 35.47 kg at age 16, reflecting an average annual increase of 3.24 kg/year. Higher percentiles (e.g., the 90th percentile in red) show an even steeper trajectory, reaching values above 57 kg by age 16. This suggests that stronger boys improve at a faster rate than their peers, with widening variability in grip strength as they age.

In Panel B (Girls), the percentile curves also show an upward trend, although the increase is more modest compared to boys. The 50th percentile line rises from approximately 14.12 kg at age 12 to 20.23 kg at age 16, averaging an annual increase of 1.53 kg/year. While all percentiles demonstrate progressive increases, the overall spread between percentiles remains narrower than in boys, indicating less variability in muscular strength among adolescent girls. These patterns confirm that: Boys outperform girls in HGS across all ages and percentiles. Growth acceleration is sharper in boys, especially between ages 14 and 15. Percentile-based normative curves are effective for identifying individuals with below- or above-average strength development. Together with the LMS-derived values in Table 5, these visual data provide a robust normative framework for evaluating physical fitness and muscular development during adolescence, supporting both individual assessments and population-level monitoring.

### 3.3 Comparison with international reference norms

**Figure 5:**
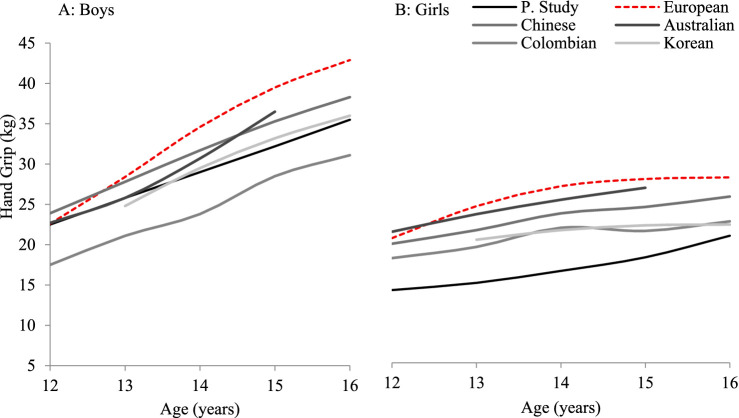
HGS (kg) at the 50th percentile (P50) in adolescent boys **(A)** and girls **(B)** aged 12–16 years, comparing the present study with published data from European, Australian, Chinese, Colombian, and Korean populations.

Figure 5 illustrates the normative reference values (50th percentile) of handgrip strength (measured in kilograms) derived from the present study in comparison with data from various international studies, including European, Australian, Chinese, Colombian, and Korean adolescent populations, disaggregated by age and gender (11, 14, 16, 41, 42). The comparison reveals that adolescent boys from South Punjab, Pakistan, generally exhibited lower median handgrip strength compared to their international counterparts. However, Pakistani boys scored consistently higher than Colombian boys across all age groups. In contrast, girls from South Punjab demonstrated lower median grip strength compared to most international samples, including Colombian girls, indicating a comparatively wider gender disparity in muscle strength.

Among the nations compared, European and Australian adolescents consistently recorded the highest median HGS values, followed by Chinese and Korean samples. The present study’s values fall below these benchmarks, highlighting the comparatively lower muscular strength development in Pakistani adolescents. Nevertheless, the trend of increasing HGS with age is consistent across all populations, reaffirming the validity of the developmental progression seen in the current cohort.

### 3.4 Status on health benefits zones for muscular strength in Pakistani adolescents

The current study utilized the handgrip strength test as a key indicator for evaluating muscular strength among adolescents. The findings are summarized in Table 6, which presents age- and gender-specific classification ranges for handgrip strength among adolescents from South Punjab, Pakistan. Table 6 provides a standardized Health Benefits Zones using a single-indicator evaluation system, categorizing handgrip strength scores into five levels: Very Poor, Poor, Medium, Good, and Excellent. These cut-off values were derived from percentile ranges, offering a structured approach for interpreting individual performance in relation to peers of the same age and gender. For boys, the handgrip strength values ranged from 8.14–11.56 kg (Very Poor) to ≥57.82 kg (Excellent) across the age spectrum. Girls, on the other hand, exhibited lower thresholds, with scores ranging from 7.52–10.08 kg (Very Poor) to ≥27.63 kg (Excellent).

**Table 6:** Age and gender-specific muscular strength as per health benefits zones.

Indicators	Gender	Age	Very Poor	Poor	Medium	Good	Excellent
Hand grip strength (kg)	Boys	12	8.14–11.56	11.57–18.63	18.64–27.03	27.04–40.21	≥40.22
		13	11.45–14.82	14.83–21.82	21.83–30.43	30.44–44.86	≥44.87
		14	14.55–17.94	17.95–24.98	24.99–33.82	33.83–49.31	≥49.32
		15	17.42–20.90	20.91–28.11	28.12–37.22	37.23–53.58	≥53.59
		16	19.96–23.63	23.64–31.17	31.18–40.66	40.67–57.81	≥57.82
	Girls	12	8.02–9.59	9.60–12.66	12.67–15.70	15.71–19.87	≥19.88
		13	7.51–9.69	9.70–13.24	13.25–16.90	16.91–21.81	≥21.82
		14	7.52–10.08	10.09–14.43	14.44–18.63	18.64–24.03	≥24.04
		15	8.81–11.63	11.64–16.14	16.15–20.23	20.24–25.23	≥25.24
		16	9.46–12.84	12.85–17.96	17.97–22.41	22.42–27.62	≥27.63

Classification thresholds are based on LMS-generated percentiles. Zones were defined as follows: Very Poor (P3–P10), Poor (P10–P35), Medium (P35–P65), Good (P65–P90), and Excellent (≥P90). This unequal distribution approach aligns with the China National Fitness Standards (2014), allowing sensitive categorization and tracking of physical performance among adolescents.

### 3.5 Status of muscular strength among Pakistani population

Table 7 presents the classification of adolescents’ muscular strength based on handgrip strength test evaluation. This system categorizes performance into five levels: Very Poor, Poor, Medium, Good, and Excellent. The results indicate that only 11.1% of adolescents fell in the Very Poor category, while 22.5% were classified as Poor. The majority of participants (37.7%) were assessed as having a Medium level of muscular strength. Additionally, 28.4% were categorized as Good, and only 9.2% achieved the excellent classification. When examined by gender, boys generally performed better than girls. Specifically, 14.6% of boys were rated Excellent compared to only 8.4% of girls. Conversely, a higher proportion of girls (22.8%) fell into the Poor category compared to boys (21.0%). These findings offer a comprehensive overview of the muscular fitness levels among 12–16-year-old adolescents in South Punjab.

**Table 7:** Status of population muscular strength as per HBZ among 12–16-year old adolescents.

HBZ	Total *n* (%)	Boys *n* (%)	Girls *n* (%)
Very Poor	329 (11.1)	148 (10.0)	181 (12.1)
Poor	669 (22.5)	328 (22.2)	341 (22.8)
Medium	970 (32.7)	553 (37.4)	417 (27.9)
Good	730 (24.6)	302 (20.4)	428 (28.7)
Excellent	272 (9.2)	146 (9.9)	126 (8.4)

HBZ, health benefit zone.

## 4 Discussion

This study established the first normative references and performance evaluation standards for absolute handgrip strength and to propose Health Benefit Zones for adolescents aged 12–16 years in South Punjab, Pakistan, using the LMS method with internal cross-validation. Consistent with international studies that have applied similar approaches (15, 18), the LMS method in our sample produced smooth age- and sex-specific percentile curves. Based on these reference values, percentile cut-offs were defined to classify individual muscular strength into performance zones referred to as Health Benefits Zones. Importantly, the reference values generated in this study are region-specific and provide a much-needed benchmark for evaluating muscular strength in this population and have significant implications for health monitoring, educational programs, and public health policy.

### 4.1 Age and sex specific trends in muscular strength

The present findings show a consistent increase in handgrip strength with age in both sexes, with boys outperforming girls across all age groups. Our results demonstrated that boys consistently exhibited higher HGS than girls across all age groups, with large effect sizes (η^2^) indicating substantial sex differences in strength variance. This is expected because androgen levels, particularly testosterone, increase markedly in boys during mid-puberty (Tanner Stages 2–4), stimulating muscle hypertrophy and improving neural recruitment (43). For example, Sun et al. (16) noted minimal sex-based differences in muscular fitness before age 11 in Chinese children, followed by accelerating gains in boys relative to girls during early teen years. Strength trajectories were similar for both sexes until approximately 12–13 years, after which boys showed a steeper increase in HGS, with the steepest gains observed between ages 14–15, aligning with the adolescent growth spurt and peak height velocity (44, 45). By contrast, girls exhibited more gradual strength gains, reflecting lower anabolic hormone levels, differences in physical activity patterns, and sociocultural factors that may limit participation in strength-enhancing activities (46). These patterns align with the findings from other populations, such as studies in China and Iran have reported minimal sex-based differences before early adolescence, followed by accelerated gains in boys through the mid-teen years (2, 16).

By age 16, boys achieved a median HGS of approximately 35 kg compared to 20 kg in girls, reflecting an average annual increase of ~3 kg/year in boys and ~1.5 kg/year in girls. Similar age- and sex-specific HGS patterns have been reported in European adolescents (30) and in the Colombian cohort (47), supporting the generalizability of these developmental trajectories. From a physiological standpoint, the greater stature, muscle cross-sectional area, and absolute fat-free mass in adolescent boys provide a biomechanical advantage in generating grip force (48). These findings are consistent with biological differences in pubertal development, though individual variations, physical activity patterns, and environmental factors may also influence strength outcomes (2, 16).

### 4.2 Age- and sex-specific anthropometric influences on handgrip strength development

The present findings demonstrate clear age- and sex-specific patterns in anthropometric growth and their relationship to handgrip strength among South Asian adolescents. Consistent with existing literature, boys in our study were generally taller and heavier than girls, factors known to correlate positively with muscular strength (2); however, by mid-adolescence boys had surpassed girls in absolute stature and mass (median height ~160 cm in 16-year-old boys vs. ~ 158 cm in girls). These growth patterns closely paralleled gains in HGS, with older adolescents exhibiting greater strength than their younger peers even after accounting for body size. Body mass showed the strongest positive association with HGS, followed by height, whereas BMI demonstrated a weaker relationship, indicating that absolute gains in lean body mass contribute more to strength than relative weight-for-height. These observed differences in body size partially explain both the sex-based disparities and international variations in grip strength, as adolescents in South Punjab, Pakistan, typically exhibit smaller stature and lighter body mass compared to their Western counterparts, contributing to relatively lower grip strength norms (15).

Anthropometric growth patterns closely paralleled the progression of HGS, with significant increases in height and body mass recorded for both sexes across the age range examined. Boys surpassed girls in absolute stature and mass by the late adolescent years (median height approximately 160 cm for boys versus 158 cm for girls at age 16). Correspondingly, absolute HGS increased significantly with age, with older adolescents demonstrating consistently greater strength compared to their younger counterparts, even after adjusting for anthropometric differences. These trends align with established biological mechanisms; during puberty, boys typically experience greater hormone-driven muscle hypertrophy, resulting in a higher proportion of lean muscle mass compared to girls, who tend to accumulate relatively more adipose tissue (43, 49, 50). This differential physiological development largely accounts for the marked sex disparities observed in HGS by mid-adolescence, reaffirming established global patterns (2, 42).

This highlights the importance of considering populations specific anthropometric profiles when establishing normative references. Overall, the results underscore that growth-related body dimensions and age together explain a substantial proportion of variance in HGS (*R*^2^ = 0.42), with boys consistently exhibiting higher absolute strength values than girls. These findings reaffirm the critical role of age, sex, and anthropometric growth patterns in shaping pediatric muscular strength development and provide valuable context for region-specific health and fitness standards.

### 4.3 Comparison with international studies

While the age and sex trends are broadly similar worldwide, the absolute magnitudes of grip strength in our cohort were lower than those reported in developed countries. By mid-adolescence, Pakistani boys in our study (age 16 median ~35 kg) fall substantially below the median grip strength of same-aged boys in Europe, China, or North America (which often exceeds 40–45 kg by ages 16–17) (11, 14, 16, 41, 42). For example, our findings align with a wide range of international research showing that handgrip strength increases steadily with age and that sex differences become more pronounced during puberty. Large-scale data from China (ages 7–18) show minimal sex differences before about 11 years, followed by a sharp divergence thereafter, with boys’ grip strength increasing by about 425% compared to a 197% increase in girls (16).

Similar developmental trajectories are reported in European studies, these studies documented a marked rise in HGS across adolescence, with boys aged 9–17 around 15.3–45.0 kg (and 13.6–28.4 kg for girls), which generally exceed the corresponding medians in our Pakistani sample at each age (51, 52). Latin American data show the same pattern: Martínez-Torres et al. and the FUPRECOL study reported consistently higher HGS in boys than girls from ages 6–17, with steeper age-related gains in boys (36). Even younger cohorts, such as those in the IDEFICS study (Europe, ages 6–10), already show boys outperforming girls in upper-limb strength, albeit at lower absolute levels (53). U. S. reference curves (NHANES) and Chinese datasets likewise show higher HGS values than those recorded in our study (14, 16). Such differences are consistent with a broader body of evidence that youth from low- and middle-income countries tend to have lower muscular fitness levels than those from high-income countries. Even in other middle-income contexts, such as Iran, adolescents show higher strength levels than South Punjab youth (2).

Taken together, these studies confirm a global pattern of progressive strength development and widening male–female differences through adolescence, a pattern also evident in our Pakistani cohort. An isolated finding showed Pakistani boys slightly outperforming Colombian boys at the median level, although Pakistani girls were notably weaker than their Colombian peers (47). This anomaly may reflect sample-specific factors or socio-cultural differences in gendered activity patterns. Overall, the evidence highlights a clear fitness gap, consistent with global surveillance reports noting that youth from low-resource regions, particularly in South Asia, tend to score lower on muscular fitness measures than peers in Europe and North America (54, 55). By situating our findings within this international context, our study not only confirms well-established developmental patterns of grip strength but also highlights the substantial gap between Pakistani youth and their international peers. These normative data contribute a crucial regional perspective to the global literature and underscore the need for targeted interventions to improve muscular fitness among adolescents in South Punjab.

### 4.4 Health benefit zones (HBZs) status

The introduction of HBZs in this study offers a practical framework for interpreting HGS performance levels. Our analysis indicated that nearly 40% of adolescents were categorized within the Very Poor or Poor zones. This finding is concerning, as low muscular strength during youth has been linked to unfavorable metabolic profiles and higher cardiovascular risk in adulthood (1, 51). Similar findings have been reported in other low- and middle-income contexts. For example, Ramírez-Vélez et al. (36) found that nearly half of Colombian schoolchildren fell into the “Needs Improvement” HBZ, moreover exhibit increased waist circumference, elevated triglycerides, and reduced cardiorespiratory fitness.

Muscular strength during adolescence is increasingly recognized as a predictor of lifelong health, with lower values associated with elevated risks of obesity, insulin resistance, and future musculoskeletal disorders (1, 51). The high prevalence of low-fitness categories in South Punjab may reflect multiple factors, including limited access to structured physical education, insufficient engagement in resistance-type activities, and widespread nutritional deficiencies that impede muscle development (56). Identifying adolescents in the very poor and poor zones underscore an urgent improvement by timely interventions, such as school-based resistance training programs have been shown to significantly enhance HGS and overall fitness (57). Thus, the HBZ framework offers actionable insight into the health status of this population.

### 4.5 Behavioral and environmental factors influencing strength

While biology sets the stage for sex differences, behavioral and environmental factors likely exacerbate the muscular strength gaps both between sexes and across populations (58, 59). In Pakistan (and South Asia more broadly), cultural norms and socioeconomic constraints can differentially shape physical activity opportunities for boys and girls (24). Adolescent boys in South Punjab may engage more frequently in sports, outdoor games, or manual labor (e.g., farming chores), all of which contribute to muscle development, whereas girls often face societal barriers to participation in vigorous physical activities (20, 23, 24). Traditional gender roles, limited access to sports facilities, and safety or modesty concerns mean that many teenage girls lead more sedentary lifestyles, which can compound their lower strength levels independent of physiology (60). Moreover, recent evidence suggests that physical inactivity and sedentary behaviors are on the rise among youth in Pakistan (61).

A study during the COVID-19 period documented a significant increase in screen time and a decline in overall physical activity in Pakistani children and adolescents (62). Such lifestyle shifts may be contributing to the generally low muscular fitness observed in our sample. Prolonged screen time and reduced participation in active play or exercise can lead to suboptimal muscle conditioning in both sexes, but especially in girls who might already have fewer opportunities for sport (63). Additionally, nutritional factors cannot be overlooked as potential explanations for the international and regional differences in strength. South Punjab is a relatively under-resourced area, and children may experience nutritional challenges (including protein deficiencies or stunting) that impede optimal muscle development (20). Chronic undernutrition or micronutrient deficiencies during childhood could lead to smaller overall body size and lower muscle mass, directly translating to weaker handgrip strength (64).

Conversely, youth from wealthier countries often benefit from better overall nutrition and health care, which support higher muscle mass and strength (65). It is also plausible that the schooling environment and physical education programs differ high-income countries typically incorporate regular PE classes and sports clubs, fostering muscle-strengthening activities, whereas many public schools in Pakistan have limited PE infrastructure. Taken together, a constellation of environmental factors diet, physical activity habits, and access to training opportunities could likely contribute to why adolescents from South Punjab trail behind their international peers in grip strength. Future research incorporating detailed dietary and activity assessments would be valuable to quantify the impact of these factors on muscular fitness in this population.

### 4.6 Implications for public health and physical education

The low levels of muscular strength identified among adolescents in South Punjab carry significant implications for both public health and physical education, particularly in resource-limited contexts. Handgrip strength is internationally recognized as a robust, low-cost indicator of overall muscular fitness and a predictor of broader health outcomes (4). Research consistently links low HGS with adverse metabolic profiles, reduced bone mineral density, elevated fat accumulation, and poor cardiometabolic health, effects that can track from adolescence into adulthood and increase the risk of chronic diseases and premature mortality (7, 66).

Our finding that nearly two in five adolescents fall into Very Poor or Poor health benefit zones is therefore concerning. This suggests that a substantial proportion of youth may be entering adulthood with compromised physical reserves, potentially contributing to future burdens of non-communicable diseases. From a public health standpoint, these results reinforce the urgency of integrating regular, structured physical activity into school curricula. Schools should prioritize PE programs that combine strength-building activities, such as resistance exercises, calisthenics, or low-cost equipment like resistance bands with aerobic components. Evidence shows that even simple, body-weight-based interventions can significantly improve muscular strength and overall fitness (57).

Furthermore, cultural considerations are essential. Creating safe, supportive environments that encourage girls’ participation can help reduce the observed gender disparities. Community-based initiatives, such as routine HGS screenings based on our HBZs and awareness campaigns, can serve as early warning systems, enabling timely referrals and tailored interventions. In settings with limited resources, these strategies represent cost-effective investments in long-term health, fostering a stronger, healthier, and more productive future generation.

### 4.7 Novelty and utility of local LMS-based standards

This study provides the first validated sex- and age-specific handgrip strength percentiles for Pakistani adolescents, generated through a rigorous LMS approach on a large, representative sample. Prior to this, practitioners in Pakistan relied on international reference data or small, fragmented local studies that did not account for regional growth patterns or body sizes. Our LMS-based centile curves address this gap by offering contextually relevant benchmarks that reflect the unique anthropometric and environmental characteristics of South Punjabi youth.

Using Western norms often misclassifies adolescents in low resource settings as unfit, even when their strength is typical for their context. By contrast, our localized reference allows pediatricians, educators, and coaches to assess an individual’s muscular strength relative to local peers. For example, a 14-year-old boy achieving 30 kg can now be placed accurately within a national percentile rather than unfairly compared to European or American standards. The “health benefit zones” derived from these normative references cutoff points further enable targeted screening, identifying those below the very poor and poor zones for early intervention, while guiding high performers to maintain their fitness levels.

Our methodological alignment with international surveillance projects (NHANES, Korean National Fitness Award) ensures comparability and credibility (42, 67). The successful cross validation via back generation further attests to the robustness of these normative references’ standard and HBZs for the intended population. Establishing this normative baseline provides a foundation for future monitoring of secular trends and informs context specific policy and intervention strategies. This study places Pakistan within the growing global network of countries with indigenous fitness reference standards, advancing equitable and relevant health assessment.

### 4.8 Strengths, limitations, and future directions

This study benefits from a large, stratified sample and the use of the internationally recognized LMS method to generate age- and sex-adjusted reference data, addressing a critical gap for South Punjab. The low mean absolute percentage error observed in cross-validation further supports the robustness of these normative standards for surveillance and intervention planning.

However, several limitations must be acknowledged. The cross-sectional design limits causal inference and precludes tracking changes in individual growth trajectories over time. Important determinants of muscular strengths such as pubertal stage, nutritional status, and socioeconomic factors were not adjusted for in the analyses, which restricts interpretation of inter individual variability. In addition, cross validation was performed only internally through a back generation test using the same dataset; no external validation cohort was available. The regional focus may also limit the generalizability of these findings to other parts of Pakistan.

Future research should adopt longitudinal designs, integrate biological and lifestyle markers, including pubertal stage, nutrition, and socioeconomic variables and evaluate targeted interventions for adolescents in lower strength categories. Examining links between muscular fitness and cognitive or academic outcomes may also provide a more comprehensive understanding of its broader health implications.

## 5 Conclusion

This study established the first age and sex specific LMS based normative reference values and Health Benefit Zones for handgrip strength among adolescents in South Punjab, Pakistan. Boys consistently demonstrated higher strength levels than girls, with the most pronounced gains occurring during mid adolescence, whereas girls exhibited a more gradual progression. These reference standards and HBZ cut offs are valuable for identifying adolescents with low muscular strength (below the 10th percentile) who may be at elevated risk for adverse health outcomes.

The provision of context specific norms enables more precise categorization of youth fitness, taking age and gender into account, and allows meaningful comparisons with international data. Importantly, these findings offer practical applications in educational and clinical settings, where routine monitoring can inform early interventions to improve strength profiles. By integrating such measures into school-based health programs and public health strategies, stakeholders can contribute to preventing obesity, cardiometabolic disorders, and other long term health risks. Overall, these data fill a critical gap in adolescent health surveillance in Pakistan and provide a robust foundation for evidence based policy and targeted fitness initiatives.

## Data Availability

The raw data supporting the conclusions of this article will be made available by the authors, without undue reservation.

## References

[1] Fraser BJ Rollo S Sampson M Magnussen CG Lang JJ Tremblay MS. Health-related criterion-referenced cut-points for musculoskeletal fitness among youth: a systematic review. Sports Med. (2021) 51:2629–46. doi: 10.1007/s40279-021-01524-8, PMID: 34339043

[2] Rostamzadeh S Saremi M Abouhossein A Vosoughi S Molenbroek JFM. Normative data for handgrip strength in Iranian healthy children and adolescents aged 7-18 years: comparison with international norms. Ital J Pediatr. (2021) 47:164. doi: 10.1186/s13052-021-01113-5, PMID: 34330318 PMC8325185

[3] Gąsior JS Pawłowski M Jeleń PJ Rameckers EA Williams CA Baran J. Test–retest reliability of handgrip strength measurement in children and preadolescents. Int J Environ Res Public Health. (2020) 17:8026. doi: 10.3390/ijerph17218026, PMID: 33142693 PMC7663254

[4] Vaishya R Misra A Vaish A Ursino N D’Ambrosi R. Hand grip strength as a proposed new vital sign of health: a narrative review of evidences. J Health Popul Nutr. (2024) 43:7. doi: 10.1186/s41043-024-00500-y, PMID: 38195493 PMC10777545

[5] Martínez-Torres J Gallo-Villegas JA Aguirre-Acevedo DC. Normative values for handgrip strength in Colombian children and adolescents from 6 to 17 years of age: estimation using quantile regression. J Pediatr. (2022) 98:590–8. doi: 10.1016/j.jped.2022.02.004, PMID: 35487284 PMC9617281

[6] Mirza F Fazal A Shabbir K Farooq H Ahmed S. Association of Grip Strength with obesity cortisol; possible indicators of biological ageing. J Int J Endorsing Health Sci Res. (2020) 8:145–52. doi: 10.29052/IJEHSR.v8.i3.2020.145-152

[7] McGrath R Johnson N Klawitter L Mahoney S Trautman K Carlson C. What are the association patterns between handgrip strength and adverse health conditions? A topical review. SAGE open Med. (2020) 8:2050312120910358. doi: 10.1177/2050312120910358, PMID: 32166029 PMC7052448

[8] Luz GD Pereira DS Minho JB Dias PDC Moraes ES da Silva VM. Association of handgrip strength with nutritional status and clinical outcomes in hospitalized pediatric patients. Clinical Nutrition ESPEN. (2024) 61:413–9. doi: 10.1016/j.clnesp.2024.04.008, PMID: 38777463

[9] Mateus-Arias OE Echeverría-Rueda M López-Páez ME Martínez-Torres J. Effects of 30-second active stretching on manual grip strength in young adults: a randomized cross-over study. Duazary. (2024) 21:285–94. doi: 10.21676/2389783X.6128

[10] Mehmet H Yang AWH Robinson SR. Measurement of hand grip strength in the elderly: a scoping review with recommendations. J Bodyw Mov Ther. (2020) 24:235–43. doi: 10.1016/j.jbmt.2019.05.029, PMID: 31987550

[11] Catley MJ Tomkinson GR. Normative health-related fitness values for children: analysis of 85347 test results on 9–17-year-old Australians since 1985. Br J Sports Med. (2013) 47:98–108. doi: 10.1136/bjsports-2011-090218 22021354

[12] Peterson MD Krishnan C. Growth charts for muscular strength capacity with quantile regression. Am J Prev Med. (2015) 49:935–8. doi: 10.1016/j.amepre.2015.05.013, PMID: 26232900 PMC4656076

[13] Perna FM Coa K Troiano RP Lawman HG Wang CY Li Y. Muscular grip strength estimates of the U.S. population from the National Health and nutrition examination survey 2011-2012. J Strength Cond Res. (2016) 30:867–74. doi: 10.1519/jsc.0000000000001104, PMID: 26196662 PMC7197498

[14] Laurson KR Saint-Maurice PF Welk GJ Eisenmann JC. Reference curves for Field tests of musculoskeletal fitness in U.S. children and adolescents: the 2012 NHANES National Youth Fitness Survey. The. J Strength Cond Res. (2017) 31:2075–82. doi: 10.1519/JSC.0000000000001678, PMID: 27741055

[15] Cohen DD Voss C Taylor MJD Stasinopoulos DM Delextrat A Sandercock GRH. Handgrip strength in English schoolchildren. Acta Paediatr. (2010) 99:1065–72. doi: 10.1111/j.1651-2227.2010.01723.x, PMID: 20178516

[16] Sun Y Yin X Li Y Bi C Li M Yang X. Normative values for muscular fitness for Chinese children and adolescents aged 7–18 years. Sustainability. (2020) 12:6078. doi: 10.3390/su12156078, PMID: 40771761

[17] Lavie CJ Carbone S Kachur S O'Keefe EL Elagizi A. Effects of physical activity, exercise, and fitness on obesity-related morbidity and mortality. Curr Sports Med Rep. (2019) 18:292–8. doi: 10.1249/JSR.0000000000000623, PMID: 31389871

[18] Bohannon RW. Test-retest reliability of measurements of hand-grip strength obtained by dynamometry from older adults: a systematic review of research in the PubMed database. J Frailty Aging. (2017) 6:83–7. doi: 10.14283/jfa.2017.8 28555708

[19] Collins J Porter J Truby H Huggins CE. How does nutritional state change during a subacute admission? Findings and implications for practice. Eur J Clin Nutr. (2016) 70:607–12. doi: 10.1038/ejcn.2016.2, PMID: 26931666

[20] Long L Hamdani SD Hamdani SMZH Zhuang J Khurram H Hadier SG. Establishing age- and sex-specific anthropometric growth references standards for South Punjab adolescents utilizing the LMS method: findings from the Pakistani population. Front Public Health. (2024) 12:1–17. doi: 10.3389/fpubh.2024.1417284 PMC11424522 39328999

[21] Fühner T Kliegl R Arntz F Kriemler S Granacher U. An update on secular trends in physical fitness of children and adolescents from 1972 to 2015: a systematic review. Sports Med. (2021) 51:303–20. doi: 10.1007/s40279-020-01373-x, PMID: 33159655 PMC7846517

[22] Omar MTA Alghadir AH Zafar H Al Baker S. Hand grip strength and dexterity function in children aged 6-12 years: a cross-sectional study. J Hand Ther. (2018) 31:93–101. doi: 10.1016/j.jht.2017.02.004, PMID: 28343852

[23] Hamdani S Zhuang J Hadier SG Khurram H. Establishment of health related physical fitness evaluation system for school adolescents aged 12-16 in Pakistan: a cross-sectional study. Front Public Health. (2023) 11:1212396. doi: 10.3389/fpubh.2023.1212396, PMID: 37829094 PMC10564982

[24] Hadier SG Liu Y Long L Hamdani SMZH Khurram H Hamdani SD. Assessment of physical literacy in 8- to 12-year-old Pakistani school children: reliability and cross-validation of the Canadian assessment of physical literacy-2 (CAPL-2) in South Punjab, Pakistan. BMC Public Health. (2024) 24:1726. doi: 10.1186/s12889-024-19185-3, PMID: 38943131 PMC11212239

[25] Hadier SG Yinghai L Long L Hamdani SD Hamdani SMZH. Assessing physical literacy and establishing normative reference curves for 8–12-year-old children from South Punjab, Pakistan: the PAK-IPPL cross-sectional study. PLoS One. (2025) 20:e0312916. doi: 10.1371/journal.pone.0312916, PMID: 39932941 PMC11813120

[26] Suresh K Chandrashekara S. Sample size estimation and power analysis for clinical research studies. J Hum Reprod Sci. (2012) 5:7–13. doi: 10.4103/0974-1208.97779, PMID: 22870008 PMC3409926

[27] Liu Y Hadier SG Liu L Hamdani SMZH Hamdani SD Danish SS. Assessment of the relationship between body weight status and physical literacy in 8 to 12 year old Pakistani school children: the PAK-IPPL cross-sectional study. Children. (2023) 10:363. doi: 10.3390/children10020363, PMID: 36832492 PMC9955071

[28] Survey, N.H.a.N.E. Anthropometry procedures manual. (2007) Available online at:https://www.cdc.gov/nchs/data/nhanes/nhanes_07_08/manual_an.pdf.

[29] Wind AE Takken T Helders PJM Engelbert RHH. Is grip strength a predictor for total muscle strength in healthy children, adolescents, and young adults? Eur J Pediatr. (2010) 169:281–7. doi: 10.1007/s00431-009-1010-4 19526369

[30] Pratt J De Vito G Narici M Segurado R. Grip strength performance from 9431 participants of the GenoFit study: normative data and associated factors. GeroScience. (2021) 43:2533–46. doi: 10.1007/s11357-021-00410-5, PMID: 34213693 PMC8599604

[31] Ramos-Sepúlveda JA Ramírez-Vélez R Correa-Bautista JE Izquierdo M García-Hermoso A. Physical fitness and anthropometric normative values among Colombian-Indian schoolchildren. BMC Public Health. (2016) 16:962. doi: 10.1186/s12889-016-3652-2, PMID: 27619491 PMC5020445

[32] Roberts HC Denison HJ Martin HJ Patel HP Syddall H Cooper C. A review of the measurement of grip strength in clinical and epidemiological studies: towards a standardised approach. Age Ageing. (2011) 40:423–9. doi: 10.1093/ageing/afr051, PMID: 21624928

[33] Zhang F Bi C Yin X Chen Q Li Y Liu Y. Physical fitness reference standards for Chinese children and adolescents. Sci Rep. (2021) 11:4991. doi: 10.1038/s41598-021-84634-7, PMID: 33654251 PMC7925655

[34] Hui SS-C Ru Z Koya S Hisashi N. Physical activity and health-related fitness in Asian adolescents: the Asia-fit study. J Sports Sci. (2020) 38:273–9. doi: 10.1080/02640414.2019.1695334 31774367

[35] China, M.o.E.o.t.P.s.R.o. Notice of the Ministry of Education on the National Student Physical Fitness Standard (revised 2014). China: Ministry of Education of the people’s republic of China Beijing (2014).

[36] Ramírez-Vélez R Morales O Peña-Ibagon JC Palacios-López A Prieto-Benavides DH Vivas A. Normative reference values for handgrip strength in Colombian schoolchildren: the FUPRECOL study. The. J Strength Cond Res. (2017) 31:217–26. doi: 10.1519/JSC.0000000000001459 27135472

[37] Zhu Z Yang Y Kong Z Zhang Y Zhuang J. Prevalence of physical fitness in Chinese school-aged children: findings from the 2016 physical activity and fitness in China—the youth study. J Sport Health Sci. (2017) 6:395–403. doi: 10.1016/j.jshs.2017.09.003, PMID: 30356643 PMC6189247

[38] Wilcox R. One-way and two-way ANOVA: inferences about a robust, Heteroscedastic Measure of Effect Size. Methodology. (2022) 18:58–73. doi: 10.5964/meth.7769

[39] Cole TJ Green PJ. Smoothing reference centile curves: the lms method and penalized likelihood. Stat Med. (1992) 11:1305–19. doi: 10.1002/sim.4780111005, PMID: 1518992

[40] Meade N. Industrial and business forecasting methods, Lewis, C.D., borough Green, Sevenoaks, Kent: Butterworth, 1982. Price: £9.25. J Forecasting. (1983) 2:194–6. doi: 10.1002/for.3980020210, PMID: 40772425

[41] Ramírez-Vélez R Rodrigues-Bezerra D Correa-Bautista JE Izquierdo M Lobelo F. Reliability of health-related physical fitness tests among Colombian children and adolescents: the FUPRECOL study. PLoS One. (2015) 10:e0140875. doi: 10.1371/journal.pone.0140875, PMID: 26474474 PMC4608730

[42] Lee S Ko BG Park S. Physical fitness levels in Korean adolescents: the National Fitness Award Project. J Obes Metab Syndr. (2017) 26:61–70. doi: 10.7570/jomes.2017.26.1.61, PMID: 31089495 PMC6484928

[43] Archibald AB Graber JA Brooks-Gunn J. Pubertal processes and physiological growth in adolescence In: Adams GR Berzonsky MD, editors. Blackwell handbook of adolescence, Malden, Massachusetts, USA: Blackwell Publishing (2003)

[44] Malina RM Rogol AD Cumming SP Silva MJC e. Biological maturation of youth athletes: assessment and implications. Br J Sports Med. (2015) 49:852–9. doi: 10.1136/bjsports-2015-094623, PMID: 26084525

[45] Malina RM. Top 10 research questions related to growth and maturation of relevance to physical activity, performance, and fitness. Res Q Exerc Sport. (2014) 85:157–73. doi: 10.1080/02701367.2014.897592, PMID: 25098012

[46] Gharahdaghi N Phillips BE Szewczyk NJ Smith K Wilkinson DJ Atherton PJ. Links between testosterone, Oestrogen, and the growth hormone/insulin-like growth factor Axis and resistance exercise muscle adaptations. Front Physiol. (2021) 11:621226. doi: 10.3389/fphys.2020.621226, PMID: 33519525 PMC7844366

[47] Ramírez-Vélez R Rincón-Pabón D Correa-Bautista JE García-Hermoso A Izquierdo M. Handgrip strength: normative reference values in males and females aged 6–64 years old in a Colombian population. Clinical Nutrition ESPEN. (2021) 44:379–86. doi: 10.1016/j.clnesp.2021.05.009, PMID: 34330493

[48] Rauch F Neu CM Wassmer G Beck B Rieger-Wettengl G Rietschel E. Muscle analysis by measurement of maximal isometric grip force: new reference data and clinical applications in pediatrics. Pediatr Res. (2002) 51:505–10. doi: 10.1203/00006450-200204000-00017, PMID: 11919337

[49] Silva DAS Martins PC. Impact of physical growth, body adiposity and lifestyle on muscular strength and cardiorespiratory fitness of adolescents. J Bodyw Mov Ther. (2017) 21:896–901. doi: 10.1016/j.jbmt.2017.01.007, PMID: 29037646

[50] Rogol AD Clark PA Roemmich JN. Growth and pubertal development in children and adolescents: effects of diet and physical activity1234. Am J Clin Nutr. (2000) 72:521S–8S. doi: 10.1093/ajcn/72.2.521S, PMID: 10919954

[51] Ortega FB Leskošek B Blagus R Gil-Cosano JJ Mäestu J Tomkinson GR. European fitness landscape for children and adolescents: updated reference values, fitness maps and country rankings based on nearly 8 million test results from 34 countries gathered by the FitBack network. Br J Sports Med. (2023) 57:299. doi: 10.1136/bjsports-2022-106176, PMID: 36623866 PMC9985767

[52] Tomkinson GR Lang JJ Rubín L McGrath R Gower B Boyle T. International norms for adult handgrip strength: a systematic review of data on 2.4 million adults aged 20 to 100+ years from 69 countries and regions. J Sport Health Sci. (2025) 14:101014. doi: 10.1016/j.jshs.2024.101014, PMID: 39647778 PMC11863340

[53] De Miguel-Etayo P Gracia-Marco L Ortega FB Intemann T. Physical fitness reference standards in European children: the IDEFICS study. Int J Obes. (2014) 38:S57–66. doi: 10.1038/ijo.2014.136 25376221

[54] Hardman K. Murphy C. Routen A. Tones S. World-wide survey of school physical education. (2013); Available online at:https://cev.org.br/media/biblioteca/229335eng_compressed.pdf.

[55] Gaziano TA Bitton A Anand S Abrahams-Gessel S Murphy A. Growing epidemic of coronary heart disease in low- and middle-income countries. Curr Probl Cardiol. (2010) 35:72–115. doi: 10.1016/j.cpcardiol.2009.10.002, PMID: 20109979 PMC2864143

[56] Hadier SG Yinghai L Long L Hamdani SD Hamdani SMZH. Mediation role of cardiorespiratory fitness on association of physical activity and physical literacy among 8–12 years old children: the PAK-IPPL cross-sectional study. Front Pediatr. (2024) 12:1383670. doi: 10.3389/fped.2024.1383670, PMID: 39346638 PMC11427255

[57] Villa-González E Barranco-Ruiz Y García-Hermoso A Faigenbaum AD. Efficacy of school-based interventions for improving muscular fitness outcomes in children: a systematic review and meta-analysis. Eur J Sport Sci. (2023) 23:444–59. doi: 10.1080/17461391.2022.2029578, PMID: 35023448

[58] Cafri G Thompson JK Ricciardelli L McCabe M. Pursuit of the muscular ideal: physical and psychological consequences and putative risk factors. Clin Psychol Rev. (2005) 25:215–39. doi: 10.1016/j.cpr.2004.09.003, PMID: 15642647

[59] Smith JJ Eather N Weaver RG Riley N Beets MW Lubans DR. Behavioral correlates of muscular fitness in children and adolescents: a systematic review. Sports Med. (2019) 49:887–904. doi: 10.1007/s40279-019-01089-7, PMID: 30864143

[60] Ganson KT Rodgers RF Nagata JM Murray SB Jones PJ Griffiths S. Problematic muscularity-oriented behaviors: overview, key gaps, and ideas for future research. Body Image. (2022) 41:262–6. doi: 10.1016/j.bodyim.2022.03.005, PMID: 35325664

[61] Sherriff A Wright CM Reilly JJ McColl J Ness A Emmett P. Age- and sex-standardised lean and fat indices derived from bioelectrical impedance analysis for ages 7–11 years: functional associations with cardio-respiratory fitness and grip strength. Br J Nutr. (2008) 101:1753–60. doi: 10.1017/S0007114508135814, PMID: 19025717

[62] Ali A Siddiqui AA Arshad MS Iqbal F Arif TB. Effects of COVID-19 pandemic and lockdown on lifestyle and mental health of students: a retrospective study from Karachi, Pakistan. Ann Med Psychol. (2022) 180:S29–37. doi: 10.1016/j.amp.2021.02.004, PMID: 33612842 PMC7883721

[63] Robertson MC Song J Taylor WC Durand CP Basen-Engquist KM. Urban-rural differences in aerobic physical activity, muscle strengthening exercise, and screen-time sedentary behavior. J Rural Health. (2018) 34:401–10. doi: 10.1111/jrh.12295, PMID: 29451333 PMC8170852

[64] Orsso CE Tibaes JRB Oliveira CLP Rubin DA Field CJ Heymsfield SB. Low muscle mass and strength in pediatrics patients: why should we care? Clin Nutr. (2019) 38:2002–15. doi: 10.1016/j.clnu.2019.04.012, PMID: 31031136

[65] Popkin BM. The nutrition transition and its health implications in lower-income countries. Public Health Nutr. (1998) 1:5–21. doi: 10.1079/PHN19980004, PMID: 10555527

[66] García-Hermoso A Cavero-Redondo I Ramírez-Vélez R Ruiz JR Ortega FB Lee DC. Muscular strength as a predictor of all-cause mortality in an apparently healthy population: a systematic review and Meta-analysis of data from approximately 2 million men and women. Arch Phys Med Rehabil. (2018) 99:2100–2113.e5. doi: 10.1016/j.apmr.2018.01.008 29425700

[67] CDC. NHANES anthropometry procedures manual 2021. (2021); Available online at:https://wwwn.cdc.gov/nchs/data/nhanes/public/2021/manuals/2021-Anthropometry-Procedures-Manual-508.pdf.

